# The effects of spoiler types on audience immersion: an inter-subject correlation study of heart rate

**DOI:** 10.1186/s41235-026-00733-x

**Published:** 2026-05-21

**Authors:** Xiaohan Zhou, Hao Chai, Wenjuan Zhang, Wuheng Zuo, Qing Liu

**Affiliations:** 1 College of Education, Zhejiang University of Technology, Hangzhou, 310014 China; 2 Mental Health Education Center, Xidian University, Xi’an, Shaanxi China

**Keywords:** Spoiler types, Narrative immersion, Heart ratesynchrony, Cognitive load, Audience engagement

## Abstract

This study used a between-subjects experimental design to examine how five spoiler conditions, including a complete scene without spoiler, a chaotic scene without spoiler, a video spoiler, a verbal spoiler, and a written spoiler, influenced viewers’ narrative engagement, heart rate synchrony measured by inter-subject correlation, and task performance while watching suspenseful film clips. Seventy-six university students were randomly assigned to one of the spoiler groups and viewed clips from the film *Revenge*. Narrative engagement was assessed using the Narrative Engagement Scale (NES), and ECG signals were recorded to derive heart rate time series for ISC-HR analyses. Key cue search task was indexed by a sticker-counting task during viewing. Results indicated that the video spoiler group showed the most consistent advantages, including higher narrative engagement, more frequent key cue search task, and the highest ISC-HR during the spoiler exposure phase. In contrast, the chaotic scene without spoiler group showed the weakest subjective engagement profile, with differences most evident in comprehension-related engagement and partially attentional focus. ISC-HR varied by spoiler modality primarily during the spoiler exposure phase, with the video spoiler group eliciting the highest synchrony, whereas ISC-HR during movie watching did not show clear group differences. Overall, these findings suggest that spoiler modality and narrative coherence shape viewers’ cognitive processing and cue monitoring during film viewing, and that ISC-HR can be used as a complementary physiological indicator to support the interpretation of immersion-related self-reports by indexing shared response dynamics among viewers.

**Supplementary Information:**

The online version contains supplementary material available at 10.1186/s41235-026-00733-x.

## Significance Statement

This study bridges the real-world challenge of how spoilers affect audience immersion in films with fundamental research on cognitive and physiological responses to narrative media. In today’s digital age, spoilers are ubiquitous on social media, often perceived as diminishing enjoyment; yet, their actual impact remains debated. By combining subjective measures (narrative engagement scales), behavioral tasks (key cue search task), and objective physiological data (heart rate  synchrony), this work reveals that how spoilers are delivered (e.g., video vs. text) profoundly shapes audience immersion, attention, and emotional resonance. As a use-inspired basic research, the study addresses the practical problem of optimizing media consumption experiences while advancing theoretical understanding of narrative processing. Key findings demonstrate that dynamic video spoilers enhance immersion by providing multimodal ‘cognitive scaffolding,’ whereas chaotic narratives disrupt engagement. This challenges assumptions that spoilers uniformly harm enjoyment and highlights the importance of narrative coherence. By uncovering the mechanisms behind spoiler effects, such as reduced cognitive load and heightened physiological synchrony, the research offers actionable insights for filmmakers, educators, and digital platforms. For example, strategically designed previews (e.g., key scene trailers) could improve audience focus without sacrificing emotional impact. Ultimately, this work transforms a common cultural frustration into an opportunity to harmonize storytelling artistry with scientific principles of attention and memory.

**Supplementary Information:**

The online version contains supplementary material available at 10.1186/s41235-026-00733-x.

## Introduction

Suspense films such as *Inception* and *Triangle* captivate audiences through intense and complex plots, leading to deep narrative immersion and temporary neglect of real-world tasks. This phenomenon exemplifies what is known as narrative transportation. Narrative transportation refers to the psychological process whereby individuals transition from reality into fictional narrative worlds (Gerrig, 1993). This immersive state reallocates attention and cognitive resources to the narrative, diminishing awareness of real-world concerns (Green & Brock, 2000). This state of immersion influences overall satisfaction, narrative consistency, and emotional resonance, and it may also be accompanied by collective neural responses during the reception of shared narratives (Grall et al., 2021a, 2021b). The prevalence of spoilers on social media platforms presents significant challenges to immersive experiences. Spoilers potentially disrupt audience engagement by revealing critical plot points in advance, fundamentally altering intended narrative journeys. They are widely perceived as diminishing viewing enjoyment (Yan & Tsang, 2016), with data from 465 Chinese films demonstrating statistically significant negative impacts on box office revenue, most pronounced within the first 6 days after release (Li et al., 2022).

Spoiler effects on immersion remain complex and sometimes contradictory. Experimental research has demonstrated that spoilers can diminish anticipation, disrupt viewing experiences, and decrease engagement (Daniel & Katz, 2019; Johnson & Rosenbaum, 2018). Replication studies confirmed that spoilers can significantly reduce story enjoyment (Johnson & Rosenbaum, 2015; Levine et al., 2016). Yet paradoxically, spoilers could enhance story enjoyment in certain contexts (Leavitt & Christenfeld, 2011). Individual differences further complicate these effects. High need for cognition individuals experienced greater enjoyment with non-spoiled stories, but this advantage disappears with spoilers (Levine et al., 2016). Spoiled endings can make participants less drawn into stories, while enabling better target task completion, suggesting spoilers may facilitate rather than impair cognitive performance (A.L.Cohen et al., 2015). When endings matched genre expectations, spoilers may decrease processing fluency, but they can also serve strategic emotion-regulation functions, as some audiences actively seek spoilers to avoid negative emotions without significantly reducing overall enjoyment or perceived suspense (Brookes et al., 2024).

Prospective memory theory provides a useful framework for examining how immersion in a narrative may interfere with the completion of intended tasks. Prospective memory refers to the ability to remember intentions for future actions, which is essential for daily life and goal-directed behavior (Hering et al., 2018; Kliegel et al., 2016; McDaniel & Einstein, 2007; Tenenboim-Weinblatt, 2013). Executing future intentions requires continuous cognitive resource allocation. Narrative immersion may interfere with prospective memory by occupying attention, as audiences overlooked monitoring keywords during coherent narrative processing (Smith et al., 2007). Spoilers may disrupt natural prospective memory processes by shifting cognitive focus from narrative progression to causal retrospective analysis. This shift alters how viewers allocate attentional resources between the narrative and concurrent tasks. In principle, this transition may enhance prospective memory performance either by reducing narrative immersion or by providing contextual cues that facilitate monitoring and alleviate cognitive load (A.L.Cohen et al., 2015).

The influence of spoilers on audience experience remains debated in psychology and media studies. Existing research largely relies on post-viewing self-reports, which are vulnerable to memory bias and limited in capturing immersion as it unfolds dynamically. Beyond methodological limitations, there is a theoretical need for measures that can reveal whether narrative content systematically coordinates physiological processes across viewers, which is a core question for understanding media as shared social experience. Neurocinematic studies demonstrate that when viewers are deeply engaged in a narrative, their brain activity shows convergent patterns, reflected in increased inter-subject correlation (ISC) (Hasson et al., 2004, 2008a). Building on this framework, inter-subject correlation of heart rate (ISC-HR) provides a dynamic physiological view of what Schmälzle et al. (2015) call collective engagement. The measure is not meant to imply literal beat-to-beat synchrony, which is usually weak in statistical terms, but to gauge the narrative’s capacity to serve as a collective pacemaker (Grall et al., 2021a, 2021b). Importantly, we treat ISC-HR as an index of relative synchrony across spoiler formats rather than as an absolute measure of immersion. Higher correlation has been interpreted as indicating that the story is constraining viewers’ attention and emotional trajectories in a similar way, leading diverse individuals through a partially shared time-locked experience (S.S.Cohen et al., 2017; Pérez et al., 2021). By indexing real-time convergence in physiological responses, ISC-HR can examine whether spoilers decouple viewers from this intended narrative arc, a nuance that static subjective measures often fail to capture. Empirical work applying ISC to spoiler research remains scarce, and the relationship between ISC-based indices and self-reported immersion has not been systematically examined. Prior studies have also rarely differentiated spoiler forms or compared their effects. As different indicators reflect distinct facets of audience experience, with physiological measures capturing automatic responses and cognitive or behavioral measures indexing deliberate involvement (Hutson et al., 2017), integrating subjective, behavioral, and physiological indicators offers a more appropriate framework for characterizing the effects of spoilers on narrative immersion. For spoiler research, ISC-HR offers unique theoretical value by revealing whether advance information alters a narrative's capacity to create shared physiological states among viewers. The theoretical importance lies not in the absolute size of the correlation, but in its sensitivity to experimental manipulations. Specifically, it can detect how different spoiler formats modulate group-level physiological synchrony with narrative content, beyond what individual self-reports can capture.

Current research indicates that the effects of spoilers on narrative experience are not uniform and likely vary based on how audiences are exposed to and cognitively process such information. However, prior research has seldom distinguished between different spoiler forms in a systematic way or combined physiological indices with self-reported immersion. To address these research gaps, the present study utilizes a multidimensional approach integrating narrative engagement scales, Key cue search task performance, and ISC-HR. Within this framework, ISC-HR is used as a physiological index of narrative control, defined as the extent to which a story elicits consistent, stimulus-driven responses across an audience (Bezdek et al., 2015). This usage focuses on shared response dynamics rather than equating ISC-HR with subjective immersion. This methodology facilitates an investigation into whether spoilers fundamentally disrupt the biological resonance characteristic of suspenseful narratives, extending the scope of inquiry beyond subjective preferences. Through a systematic comparison of five spoiler formats, this research evaluates how different modes of information disclosure modulate the capacity of a narrative to command a convergent physiological state.

Using a single-factor between-subjects design, this study systematically investigated how five experimental conditions affected narrative immersion, target task performance, and ISC-HR in suspense film audiences. The five conditions were a complete scene without spoiler, a chaotic scene without spoiler, a video spoiler, a verbal spoiler, and a written spoiler. Grounded in narrative transportation theory and prospective memory theory, this study employs narrative engagement scales, key cue search task, and ISC-HR measurements to elucidate how spoiler types modulate psychological and physiological response mechanisms, directly addressing scholarly debates surrounding spoiler effects. We proposed specific hypotheses alongside exploratory research questions to guide the study. First, we hypothesized that spoilers, especially in rich formats like video, would serve as cognitive supports that enhance narrative comprehension and allow viewers to better manage concurrent tasks. Second, we predicted that video spoilers would produce stronger physiological resonance during spoiler exposure than written or verbal descriptions due to their multimodal nature. Third, we explored how narrative structure disruption and different spoiler formats would affect the balance between immersion and concurrent task performance, as existing research provided limited guidance for these comparisons. Finally, we investigated the degree of consistency between real-time physiological measures and retrospective self-reports. This approach provides a rigorous assessment of the mechanisms by which information foreknowledge and narrative structure reshape the cinematic experience.

## Methods

### Participants

This study recruited 76 university students, including six men and 70 women, aged 19 to 26 years (*M* = 21.72, *SD* = 1.50). Participants were randomly assigned to one of five experimental groups: 16 to the complete scene without spoiler group, 14 to the chaotic scene without spoiler group, 16 to the video spoiler group, 15 to the verbal spoiler group, and 15 to the written spoiler group. For clarity, these groups are hereafter referred to as the complete scene group, chaotic scene group, video spoiler group, verbal spoiler group, and written spoiler group, respectively. All participants were Chinese native speakers. They had normal vision and hearing, basic reading comprehension ability, and no history of cardiovascular disease or diagnosed mental disorders. Each participant provided informed consent before the experiment and received compensation after participation.

### Research instruments

#### Narrative engagement scale

To assess the participants’ film viewing experience, the Narrative Engagement Scale (NES) (Busselle & Bilandzic, 2009) was used to measure their narrative engagement. The NES was a popular scale with high reliability and validity, effectively measuring aspects such as emotional involvement and attention allocation in the audience’s narrative experience. In this study, the NES scale was found to have a reliability value of 0.83, indicating that the scale’s results had high stability and reliability. That is, the results obtained by subjects using this scale at different times or in different contexts were relatively consistent. The scale further revealed a validity score of 0.74, suggesting that it could effectively measure the intended content of the study, namely, the narrative engagement of the audience and could accurately reflect the effect of different types of spoilers on the audience’s sense of immersion.​

The NES scale comprised 12 items organized into four distinct sub-dimensions:​

*(1)Narrative Understanding,* which assessed individuals’ comprehension of narrative content, encompassing their grasp of story plots, character motivations, and event logic;​

*(2)Attentional Focus,* which reflected the degree of concentration individuals maintained while viewing or reading narrative content, as well as their susceptibility to distraction;​

*(3)Emotional Engagement,* which involved the emotional resonance between audiences and narrative characters, including both empathy and sympathy responses; and​

*(4)Narrative Presence,* which represented the subjective experience of individuals’ immersion in the narrative world and their detachment from the real-world environment.​

Each dimension contained three items measured on a 7-point scale, where 1 indicated “completely disagree” and 7 indicated “completely agree.” Higher scores signified that the description more closely aligned with the individual’s experience. The total score for narrative engagement was calculated by summing the scores from all sub-dimensions. A higher total score indicated a greater level of narrative engagement, reflecting a stronger sense of immersion.

#### Video materials

Building upon existing research that has validated the effectiveness of film clips as experimental materials (A.L.Cohen et al., 2015), this study selected a segment (01:10:50–01:27:50) from Johnnie To's Cantonese film *Vengeance* as the experimental material, with Chinese subtitles added. This video segment is approximately 17 min long and is situated at a pivotal turning point in the narrative. The plot of this segment unfolds as follows: Gauldel seeks revenge on Mr. Fung. A trap is set by DuoZaipo, who has children mark Mr. Fung with stickers. Despite his amnesia, Gauldel tenaciously pursues the culprit and ultimately identifies and shoots Mr. Fung, based on the stickers and bullet holes (See Appendix A for a detailed description of this segment). It encompasses suspenseful chase sequences, captivating elements of mystery, and subtle interactions between complex character relationships. To process the material, a high-definition original version of the film was obtained through official channels. The target segment was then extracted, ensuring the quality and integrity of the clip. Accurate counting of these cues was hypothesized to reflect the allocation of cognitive resources: immersion, which prioritizes holistic plot experience, may reduce attention to such details, whereas disruptions to immersion might free resources for cue tracking. This task was anchored in a naturally occurring visual element of the film—specifically, participants were instructed to count the number of times stickers appeared on the villain Mr. Fung’s clothing during viewing.

Notably, these stickers were not artificially inserted in post-production but constituted an integral narrative device in the original footage: as the linchpin for Gauldel’s identification of Mr. Fung (central to resolving his amnesia-driven confusion), their repeated appearance is inherently tied to viewers’ natural cognitive processing of the plot, audiences would intuitively attend to such critical cues when following the story. This inherent narrativity ensured the task’s ecological validity, as counting aligned with the natural attention patterns involved in comprehending the narrative, rather than imposing an artificial cognitive load. To standardize counting, participants received clear instructions: “Count all instances where the stickers on Mr. Fung’s clothing are visible, even briefly, regardless of camera shot type (close-ups, medium shots, or long shots).” The number of occurrences ranged from 28 to 40 times, consistent with variations in shot framing (e.g., longer shots partially obscuring the stickers). Based on collaborative discussion, the researchers segmented the original video into eight narrative scenes, each composed of approximately 57 distinct shots. For the chaotic scene group, these shots were randomly re-ordered to disrupt the original narrative sequence while preserving the visual content. This manipulation produced a temporally incoherent version of the story that differed from the chronological presentation in other groups. The detailed shot sequence for the chaotic versions is provided in Appendix B. This scene rearrangement method has been employed in prior research to alter narrative structure (Hasson et al., 2008a, 2008b).

The spoiler materials were structured as follows: participants in the video spoiler group viewed highlight clips extracted from the original video, accompanied by brief textual descriptions, before watching the complete segment. Participants in the verbal spoiler group received a concise summary of the original plot delivered at a moderate speech rate by an AI-generated voice. Participants in the written spoiler group read the same spoiler content in written form. Although the content was consistent across spoiler conditions, the mode of presentation differed.

#### Physiological multichannel instrument

In this experiment, the MP150 physiological multichannel recorder (16 channels) from Biopac Systems, USA, was used to record the subjects’ electrocardiogram data. The physiological multichannel recorder consisted of three components: the main unit, amplifier, and transducer. The primary module used in this experiment was the ECG100C electrocardiogram amplifier, with a sampling frequency of 1000 Hz and operated on a computer equipped with the Windows 10 Professional operating system, which had AcqKnowledge software installed and was connected to the physiological multichannel recorder. Throughout the experiment, subjects were required to attach electrode patches to their left and right ankles and right wrist and maintain a fixed posture to facilitate the recording of electrocardiogram data.

#### Psychopy program

The experiment utilized PsychoPy 2023.2.3 software for programming and executing the experimental procedures. PsychoPy, developed by psychologists at the University of Nottingham, was a free cross-platform software package based on the Python programming language. It could construct and execute various types of psychological experiments. Its advantage lay in its ability to precisely control the timing and manner of stimulus presentation, making it suitable for research requiring high temporal precision. In the present experiment, its extensive stimulus presentation functions, such as visual and auditory stimulus modules, were employed to meet the needs of experiments with different participant groups, ensuring a smooth experiment and effective collection of data.

### Experimental procedure

#### Preparations before the experiment

Before the experiment, the physiological signals of the subjects were collected using a physiological polygraph. The measurement of these signals relied on electrode patches, which were attached to specific parts of the participant’s body. These patches were connected to electrode clips via metal protrusions, allowing for the accurate capture of the physiological parameters. To obtain clear and stable electrocardiogram signals, the electrode patches were placed above the wrist of the right forearm and above the ankles on the inner sides of both legs. Subjects were first asked to sit quietly and rest while the experimental procedure was explained to them, before they were informed that the experiment was about to start. The experimenter then attached the heart rate-measuring electrode patches to the participants’ skin and calibrated the equipment.

#### Experiment begins

**Fig. 1:**
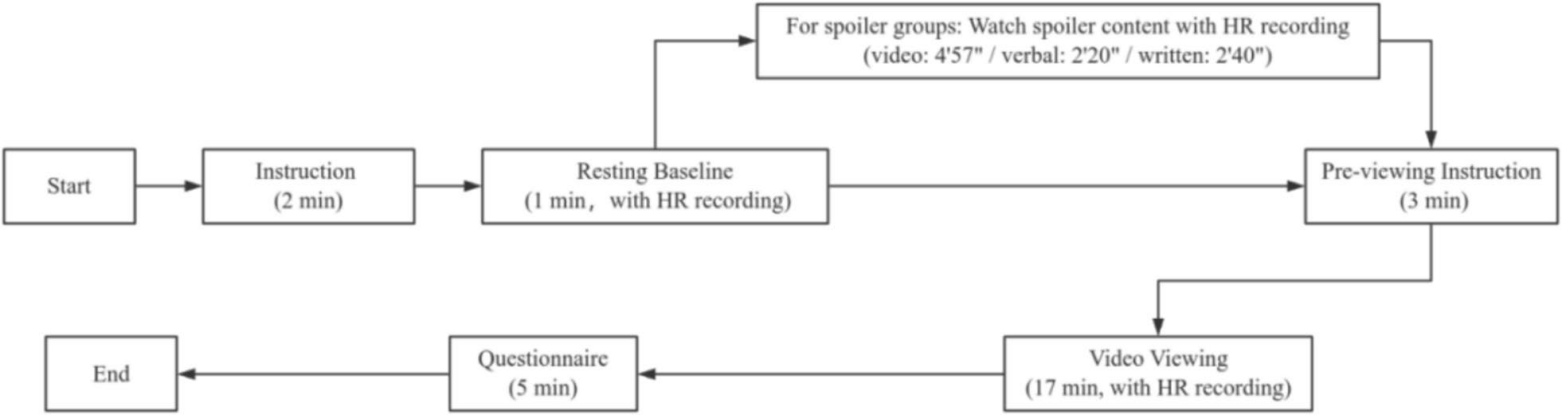
Experimental flowchart

After the formal start of the experiment, participants were first presented with a 2-min instruction by the experimenter, followed by a 1-min resting period during which their baseline heart rate was collected. Participants in the video spoiler  group, verbal spoiler  group, and written spoiler  group then received spoiler information lasting 4 min and 57 s, 2 min and 21 s, and 2 min and 40 s, respectively. Those in the complete video without spoilers group and the chaotic video without spoilers group did not receive any spoiler information and proceeded directly to the next step. Immediately afterward, all participants watched another 3-min instruction and rested. Subsequently, they engaged in a 17-min viewing activity. After the viewing session, participants were required to complete a questionnaire within 5 min. For the specific process, see Fig. 1.

### Data analysis

Electrocardiogram signals were continuously recorded throughout the experiment and preprocessed in AcqKnowledge. Signals were detrended using a 0.5 Hz high-pass filter, and line noise was attenuated using a 50 Hz notch filter. R-peaks were detected using the MarcusVollmer MATLAB toolbox, and instantaneous heart rate in beats per minute was derived from inter-beat intervals. Heart rate time series were exported to R (v4.5.0) for ISC analyses. Following the ISC-HR pipeline described by Pérez et al. (2021), duplicate timestamps were averaged when present, and each time series was linearly interpolated to a common 1 Hz temporal grid before inter-subject correlations were computed. As a first step, all raw recordings were imported into R and band-pass filtered to retain heart rate values between 50 and 180 bpm. This range excludes physiologically implausible beats and motion-induced spikes without further assumptions. The filtered series served as the input for all subsequent inter-subject correlation analyses.

ISC-HR was computed within each experimental group as an index of synchronization in heart rate fluctuations across participants during a predefined epoch. For the movie viewing phase, analyses were based on a fixed 7-min window from minutes 5 to 12 of the shared film segment. To ensure temporal comparability, each participant’s movie viewing HR series was re-zeroed to the start of the movie phase, interpolated to the common 1 Hz grid, and then analyzed over the identical 300–719 s window across all five groups.

For the formal ISC-HR analyses, Pearson's correlations were computed for all unique participant pairs within each group. These pairwise coefficients were Fisher-z transformed and averaged, and the resulting value was converted back to the correlation scale to obtain group-level ISC-HR. In parallel, participant-level ISC-HR values were derived as each participant’s mean Fisher-z–transformed correlation with all other participants in the same group and were used in subsequent inferential analyses. To assess whether the observed group-level synchrony exceeded chance levels, permutation tests with 5000 iterations were conducted by circularly shifting each participant’s time series before recomputing ISC-HR.

ISC-HR during the spoiler phase was estimated using two complementary approaches. In the primary analysis, each participant’s spoiler phase HR series was aligned to a shared temporal grid before correlations were computed, consistent with the common-time-grid approach described by Pérez et al. (2021). As a robustness check, we also computed full-duration spoiler ISC-HR using the original spoiler phase series without temporal standardization, while restricting the analysis to the common within-group overlap available for each spoiler condition. In both cases, ISC-HR was calculated using the same pairwise Pearson's correlation and Fisher-z aggregation procedure described above.

To quantify phase-related changes in synchrony, ΔISC-HR was computed at the participant level as the difference between movie viewing ISC-HR and spoiler phase ISC-HR, with positive values indicating stronger physiological synchronization during movie viewing than during spoiler exposure:

$$\Delta {\mathrm{ISC}} - {\mathrm{HR}} = {\mathrm{ISC}} - {\mathrm{HR}}_{{{\mathrm{movie}}}} - {\mathrm{ISC}} - {\mathrm{HR}}_{{{\mathrm{spoiler}}}}$$

Given that ISC-HR based on individual cardiac time series inherently produces smaller correlation magnitudes, the interpretation of our findings relies on the comparative variance across groups. By maintaining a consistent computational pipeline, we emphasize the shifts in synchrony across different spoiler modalities and movie watching phases rather than the absolute numerical thresholds of the coefficients.

**Fig. 2:**
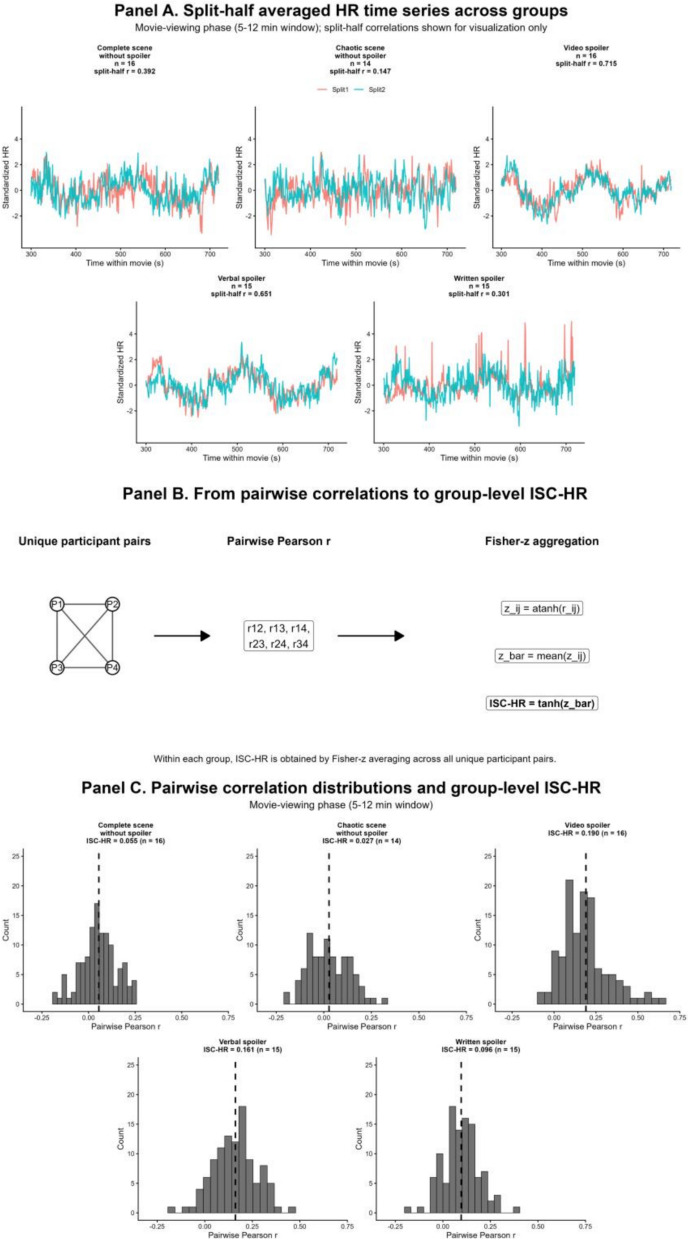
Explanation of ISC-HR calculation method

Figure 2 provides a visual summary of the ISC-HR pipeline. Figure 2A presents split-half averaged HR time series for all five groups during the movie viewing phase. Following Schmälzle et al. (2026), participants within each group were randomly divided into two halves, and the interpolated HR series were averaged within each half to visualize shared temporal fluctuations more clearly. These split-half correlations were used for visualization only and not for inferential statistics. Figure 2B illustrates the computational pathway from within-group pairwise Pearson's correlations to group-level ISC-HR. Figure 2C shows the distribution of pairwise correlations within each group, with dashed lines indicating the corresponding group-level ISC-HR values derived from Fisher-z aggregation. This visualization strategy follows Schmälzle et al. (2026) in using split-half plots for signal inspection while retaining pairwise ISC as the basis for formal statistical inference.

## Results

### Effects of different types of spoilers on participants’ ISC-HR

ISC-HR was computed for the resting, spoiler exposure, and movie watching phases as a physiological index of shared response dynamics among viewers. Higher ISC-HR values reflect stronger synchronization of heart rate fluctuations at the group level (*F*_(4,71)_ = 0.975, *p* = 0.427, *η*^*2*^ = 0.053). Baseline ISC-HR during the 1-min resting period showed no significant differences across groups: complete scene (*M* = 0.027 *SD* = 0.067), chaotic scene (*M* = 0.034, *SD* = 0.057), video spoiler (*M* = 0.026, *SD* = 0.044), verbal spoiler (*M* = 0.056, *SD* = 0.030), and written spoiler (*M* = 0.022, *SD* = 0.057), confirming comparable initial physiological states across groups and indicating that subsequent differences could not be attributed to pre-existing group differences in cardiac synchrony.

After verifying the ISC-HR during rest, we further analyzed the level of heart rate synchronization during the exposure stage of the spoilers under three groups (video, verbal, written) to explore the impact of different plot revelation forms on the synchrony of physiological responses in the test subjects. ISC-HR was computed on a stimulus-locked, length-matched time window: Each participant’s time series was aligned to the onset of spoiler exposure (*t* = 0), interpolated to 1 Hz, and ISC-HR was calculated within the common 141s window shared by all three groups. Participants in the video spoiler group showed the highest ISC-HR (*M* = 0.044, *SD* = 0.032), significantly outperforming the verbal (*M* = 0.002, *SD* = 0.035) and written (*M* = 0.021, *SD* = 0.040) groups, For full details, see Table 5 in Appendix C. To determine if these differences were statistically significant, a one-way ANOVA was performed. As shown in Table 1, the analysis confirmed that the spoiler presentation mode had a significant effect on ISC-HR (*F*_(2, 43)_ = 5.34, *p* =.008, *η*^*2*^ = 0.200).

**Table 1:** One-way ANOVA results for ISC-HR during spoiler presentation phase

Source	*SS*	*df*	*MS*	*F*	*p*	*η*^*2*^
Between groups	0.014	2	0.007	5.380	.008^**^	0.200
Within groups	0.056	43	0.001			
Total	0.070	45				

The significance levels in the table are denoted as follows:^**^p < 0.01

These results suggest that different formats trigger varying degrees of shared physiological response, with the video format inducing more robust ISC-HR across the group than the two unimodal formats. Tukey HSD tests (Table C2) showed that the video group produced significantly higher ISC-HR than the verbal group (*MD* = 0.042, *p* =.006, Cohen’s *d* = 1.254; Hedges’ *g* = 1.379), whereas the video–written contrast was not significant (*MD* = 0.019, *p* =.325, *d* = 0.637; *g* = 0.620), nor was the verbal–written contrast (*MD* =  − 0.024, *p* =.182, |*d*|= 0.506; |*g*| = 0.492). Bonferroni-adjusted pairwise t-tests (see Appendix C Table 7) corroborated this result, with only the video–verbal comparison reaching significance (*p* =.006, |*d*|= 1.067), while the video–written (*p* =.076, |*d*|= 0.660) and verbal–written comparisons (*p* =.274, |*d*|= 0.407) remained non-significant. As a robustness check, we recomputed ISC-HR using the full group-specific duration; the overall ordering was consistent with the main analysis, and the video spoiler group remained higher than the verbal and written groups (see Appendix D).

ISC-HR during the movie watching phase showed no clear group differences. A one-way ANOVA with spoiler type as the between-subjects factor yielded a non-significant main effect (*F*_(4, 71)_ = 0.507, *p* =.731, *η*^*2*^ =.028). Mean ISC-HR values during the common movie segment were closely clustered across the five spoiler groups. Tukey’s HSD comparisons did not indicate any reliable pairwise differences.

### Analysis of ΔISC and the phase with spoiler-type interaction

To investigate the shift in cardiac synchrony from the spoiler exposure phase to the movie watching phase, we calculated the ΔISC-HR index for the three spoiler modality groups. Descriptive statistics revealed a notable negative ΔISC-HR in the video spoiler group (*M* =  − 0.045, *SD* = 0.033), ΔISC-HR values for the verbal (*M* =  − 0.001, SD = 0.045), and written (*M* = − 0.018, *SD* = 0.039) groups remained near zero. A one-way ANOVA (Table 2) confirmed a significant main effect of spoiler modality on ΔISC-HR (*F*_(2, 43)_ = 11.911, *p* < .001, *η*^*2*^ =.356). Post hoc comparisons using Tukey’s HSD revealed that the decrease in synchrony for the video spoiler group was significantly greater than both the verbal spoiler group (*Δ* = − 0.100, *p* < .001) and the written spoiler group (*p* = .001). In contrast, the difference between the verbal and written groups did not reach statistical significance (*p* = .946).

**Table 2:** One-way ANOVA results for ΔISC-HR

Source	*SS*	*df*	*MS*	*F*	*p*	*η*^*2*^
Between groups	0.015	2	0.008	5.009	.011^*^	0.188
Within groups	0.065	43	0.002			
Total	0.080	45				

The significance levels in the table are denoted as follows:^*^ p <.05

To ensure the stability of these findings, a non-parametric Kruskal–Wallis test was conducted as a robustness check. The results corroborated the parametric analysis, *H*_(2)_ = 12.945, *p* = .002, with a large effect size (*η*^*2*^ = 0.255). Subsequent Dunn’s post hoc tests with Bonferroni correction confirmed that the video modality elicited a significantly larger phase-based change compared to both verbal (*p*_adj_ = 0.006) and written modalities (*p*_adj_ = 0.006). Collectively, these results demonstrate that video spoiler induce a uniquely potent reduction in inter-subject cardiac synchrony when transitioning from the spoiler to the actual film.

**Fig. 3:**
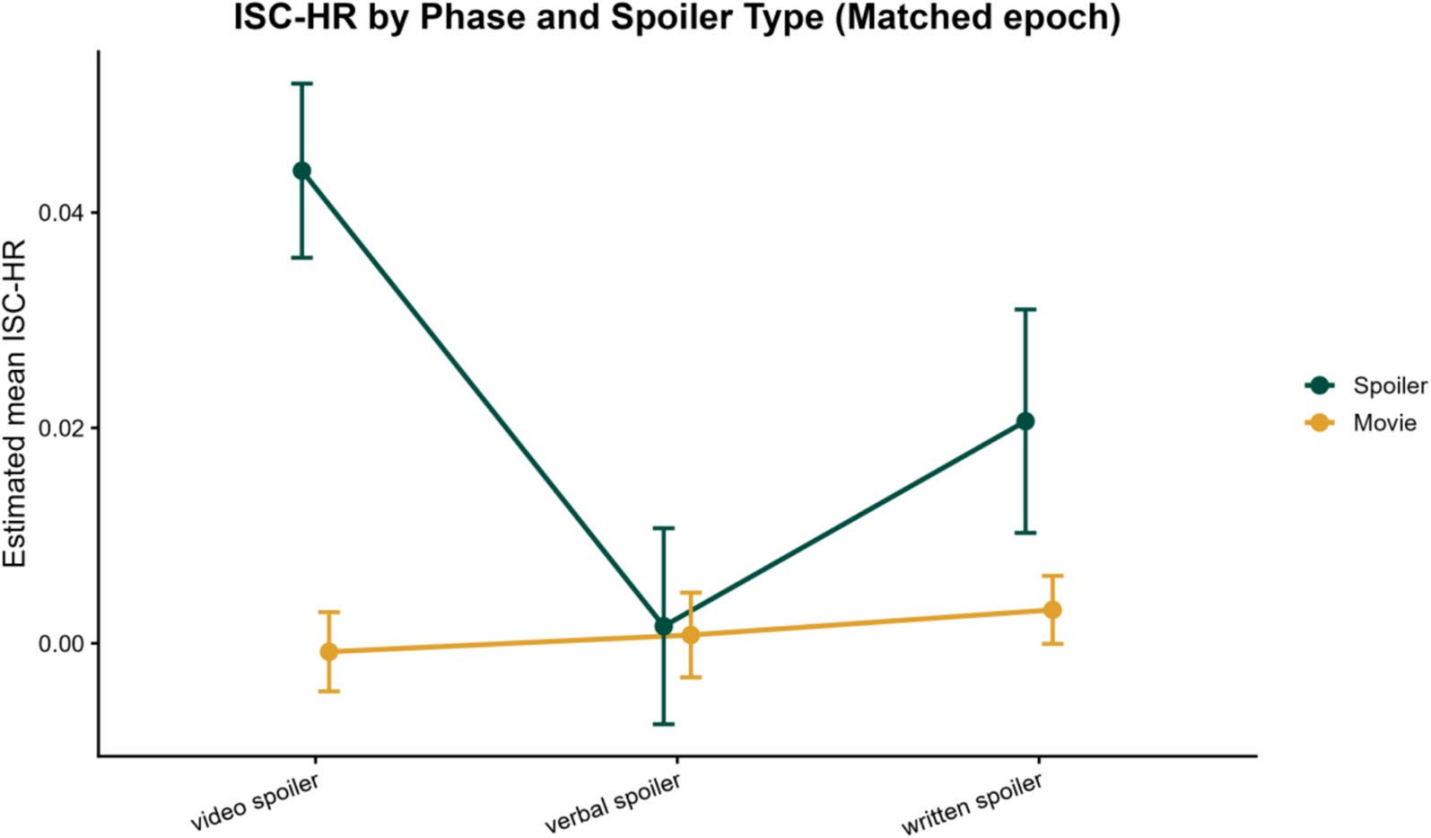
Interactive effect of spoiler modality and phase for ISC-HR

A 2 (Phase: Spoiler, Movie) × 3 (Spoiler Type: Video, Verbal, Written) mixed-design ANOVA was conducted on the ISC-HR. The analysis revealed a significant main effect of Spoiler Type (*F*_(2, 43)_ = 4.38, *p* =.019, *η*^*2*^_*P*_ = 0.091), and a significant main effect of Phase (*F*_(1, 43)_ = 13.32, *p* < 0.001, *η*^*2*^_*P*_ =.136), indicating overall variations in ISC-HR across different modalities and experimental stages. Meanwhile, the interaction effect between phase and spoiler type is significant (Fig. 2), *F*_(2, 43)_ = 5.01, *p* = .011, *η*^*2*^_*P*_ = 0.106. This interaction suggests that the influence of spoiler modality on ISC-HR was significantly moderated by whether participants were in the spoiler or the movie watching phase (Fig. 3).

**Fig. 4:**
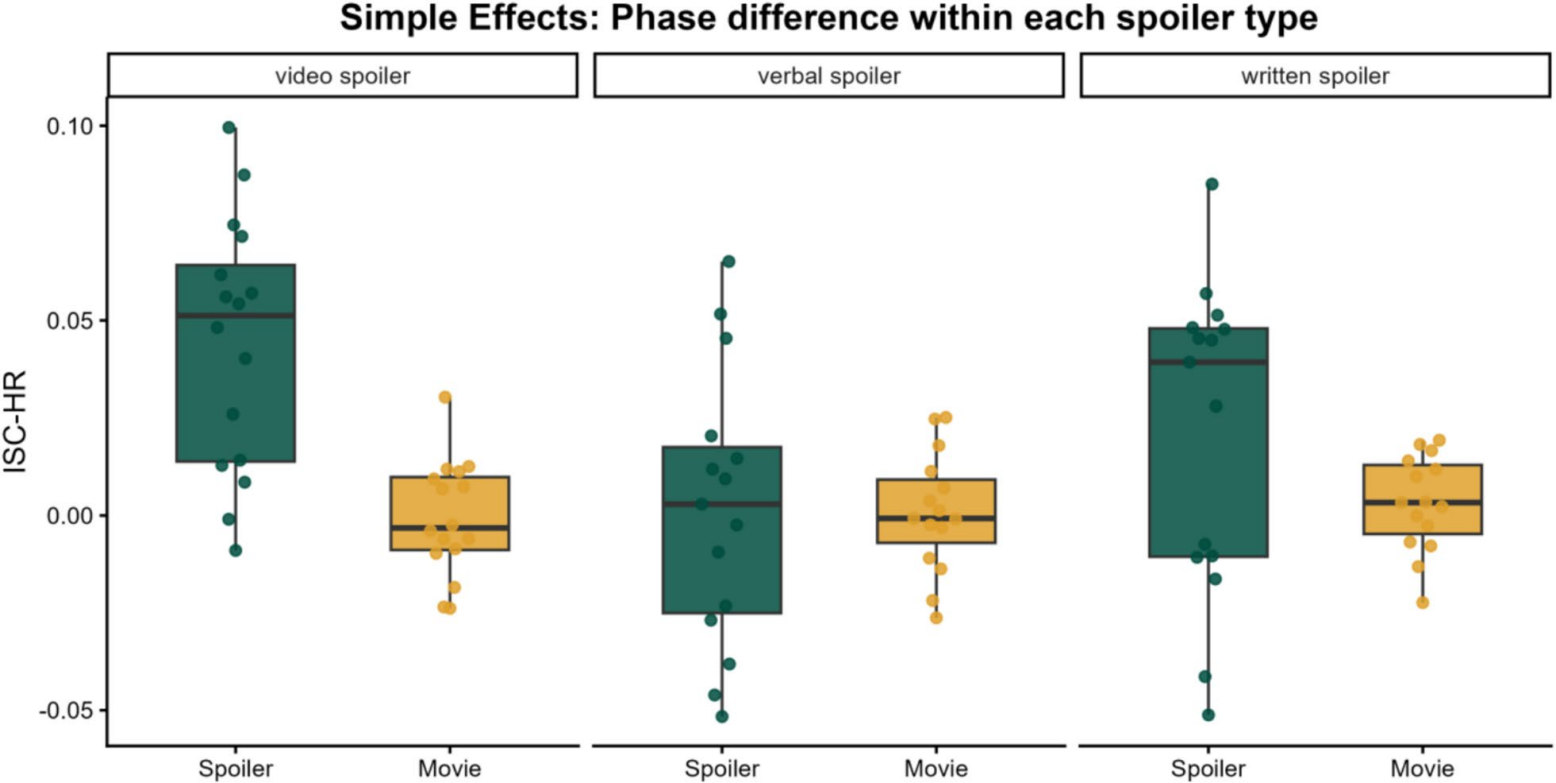
Simple effect of spoiler modality and phase for ISC-HR

To further elucidate the interaction, simple effects analyses were performed. During the Spoiler Phase, the effect of modality was prominent: pairwise comparisons revealed that the video spoiler group (*M* = 0.044, *SE* = 0.009) achieved significantly higher ISC than the verbal spoiler group (*M* = 0.002, *SE* = 0.009, *p* =.006), while no other significant between-group differences were found (*p*_s_ >.150). Conversely, during the Movie Phase, ISC levels converged across all groups, with no significant differences observed (*p*_s_ = 1.00). Within-group comparisons across phases further showed that only the video spoiler group experienced a marked decline in ISC from the spoiler to the movie stage (Δ*M* = 0.045, *t*_(43)_ = 4.58, *p* <.001) (Fig. 4).

### Analysis of key cue search frequency

**Fig. 5:**
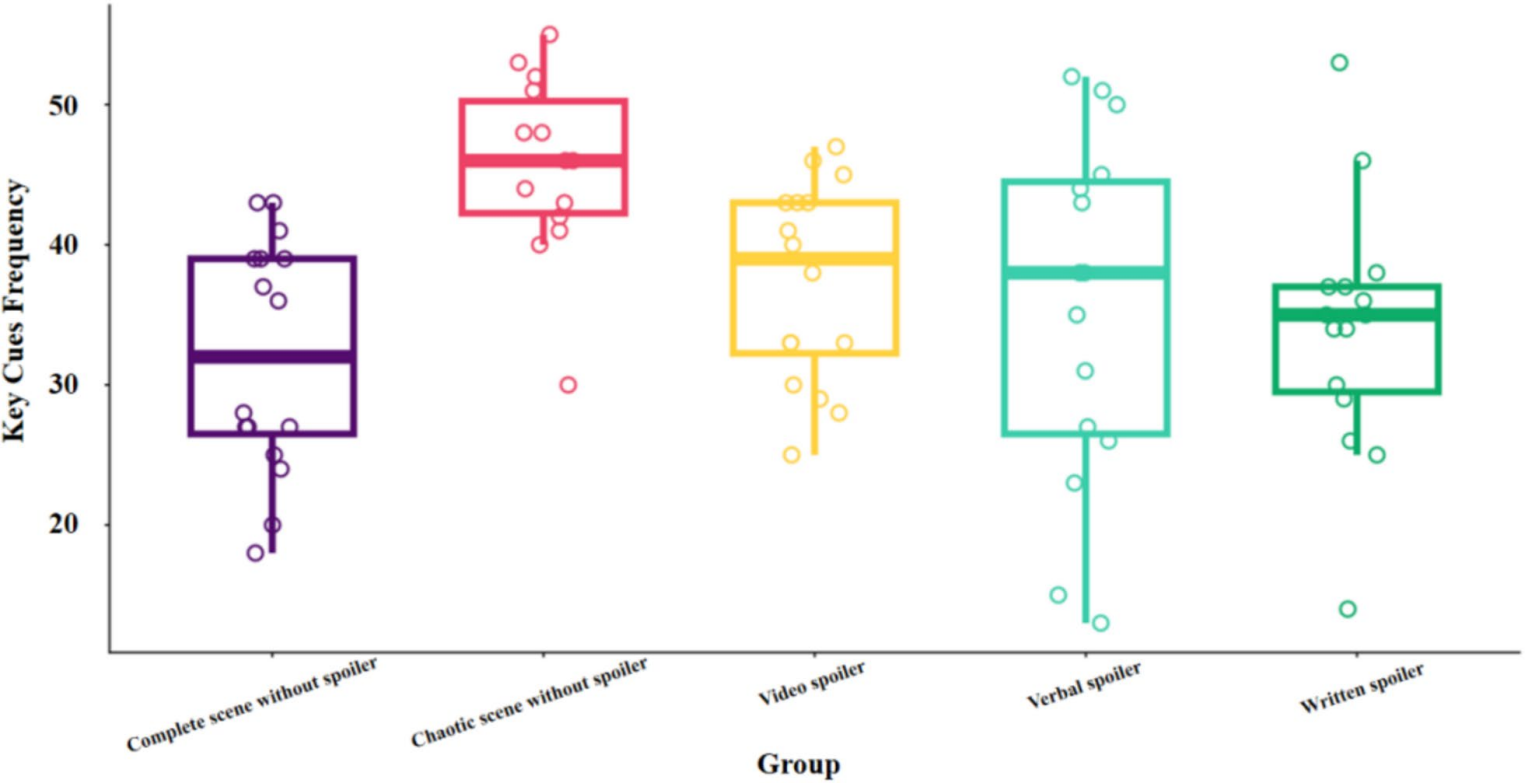
Box plot showing average search frequency for key cues across groups

Figure 5 presents the distribution of key cue search frequency across the five groups. Descriptive statistics indicated that the chaotic scene without spoiler group showed the highest sticker count (*M* = 45.643, *SD* = 6.464). The remaining groups showed lower frequency, including the video spoiler group (*M* = 37.312, *SD* = 7.134), verbal spoiler group (*M* = 35.400, *SD* = 12.591), written spoiler group (*M* = 33.933, *SD* = 8.988), and complete scene without spoiler group (*M* = 32.062, *SD* = 8.386). A one-way ANOVA revealed a significant effect of group on key cue search frequency (*F*_(4, 71)_ = 5.003, *p* =.001, *η*^*2*^ = 0.220, *ω*^*2*^ = 0.174). Tukey HSD post hoc tests showed that the chaotic scene group detected significantly more key cues than the complete group (Δ*M* = 13.580, 95% *CI* [4.392, 22.768], *p* < .001), the verbal spoiler group (Δ*M* = 10.243, 95% *CI* [0.913, 19.573], *p* = .024), and the written spoiler group (Δ*M* = 11.710, 95% *CI* [2.380, 21.039], *p* = .007). No other pairwise comparisons were significant after adjustment (*p*_s_ ≥ .094). As a robustness check, we additionally conducted Dunn test with Bonferroni correction post hoc analysis. The pattern of results converged with the ANOVA-based conclusions by confirming significant differences between the chaotic group and the complete group (*p*_adj_ < .001) as well as between the chaotic group and the written group (*p*_adj_ = .005).

### Analysis of narrative engagement

One-way ANOVAs were conducted to evaluate intergroup differences across the four dimensions of the NES and the total score. The analysis revealed a significant main effect of group on the total NES score, *F*_(4, 71)_ = 3.36, *p* = .014, *η*^*2*^ =.159, and a highly significant effect on narrative comprehension, *F*_(4, 71)_ = 8.02, *p* < .001, *η*^*2*^ =.311. The effect on attention focus reached marginal significance, *F*_(4, 71)_ = 2.45, *p* = .054, *η*^*2*^ =.121, whereas no significant differences were observed for emotional engagement (*p* = .216) or narrative presence (*p* = .846). These results suggest that spoiler modality primarily influenced the cognitive structural understanding of the narrative rather than the subjective sense of "being there" or emotional resonance.Post hoc comparisons using Tukey’s HSD further elucidated these patterns. For narrative comprehension, the video spoiler group (*M* = 17.25) scored significantly higher than the chaotic scene without spoiler group (*M* = 12.00, *p* < .001), the verbal spoiler (*M* = 13.25, *p* = .001), and the complete scene without spoiler group (*M* = 14.33, *p* = .030). The written spoiler group (*M* = 15.67) also demonstrated superior comprehension compared to the chaotic scene without spoiler group (*p* = 0.009). Regarding the total NES score, both the video spoiler group (*M* = 65.50) and written spoiler group (*M* = 62.13) groups reported significantly higher overall engagement than the chaotic scene without spoiler group (*M* = 53.07, *p*_s_ < .050). For attention focus, the video spoiler group (*M* = 16.25) showed significantly higher focus than the chaotic scene group (*M* = 13.29, *p* = .032). The power for this analysis was 0.88, confirming sufficient sensitivity to detect the differences in subjective narrative engagement across groups.

### Correlation analysis

**Fig. 6:**
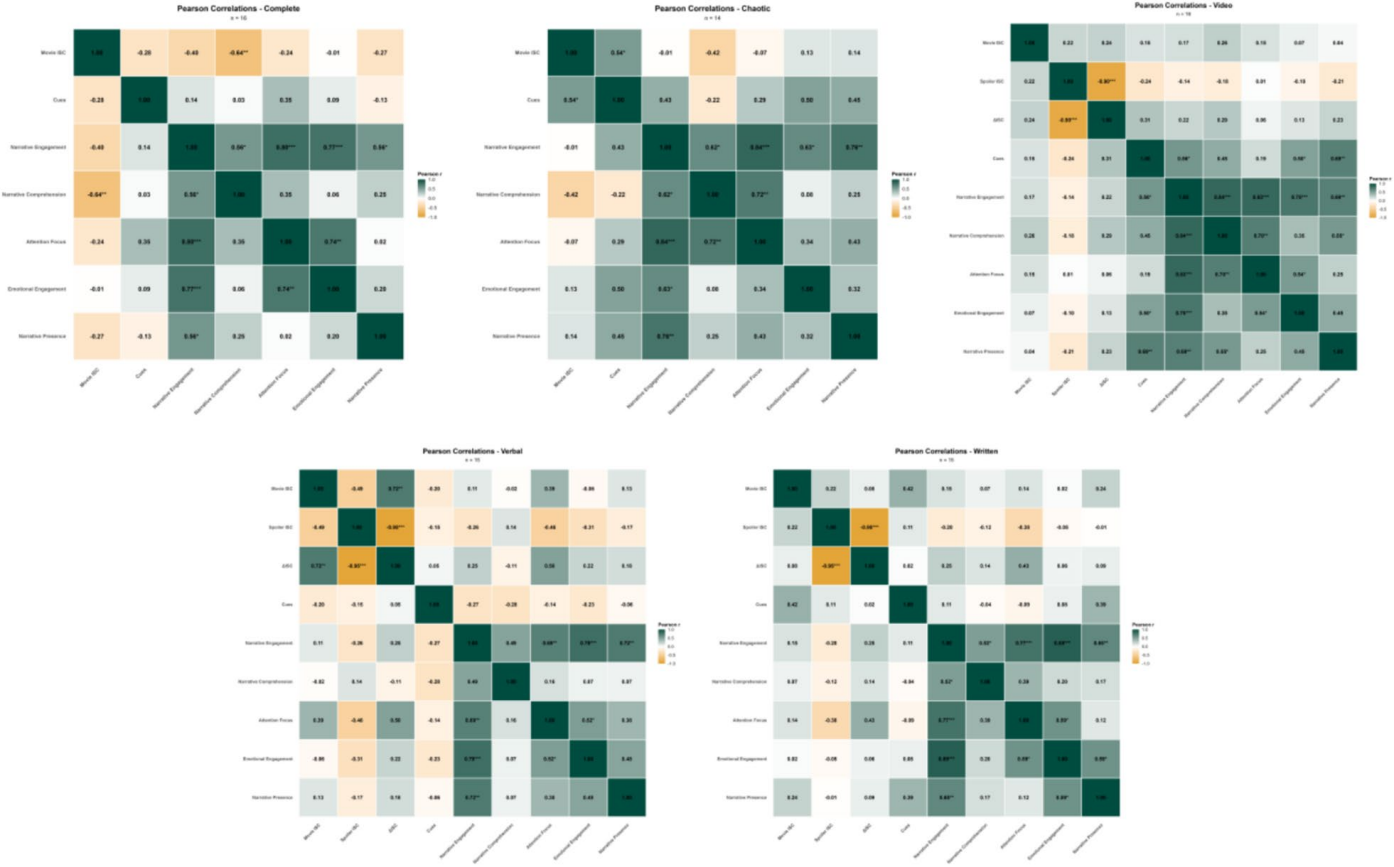
Heatmap of the correlations among dependent variables in each group. *Note:* Pearson's correlation heatmaps for each experimental group. Cell values indicate Pearson’s* r* (asterisks denote significance levels). Colors represent a diverging scale centered at zero: green indicates positive correlations, orange indicates negative correlations, and white indicates correlations close to zero

Figure 6 presents within-group Pearson's correlation heatmaps among ISC-HR indices (Spoiler ISC-HR., Movie ISC-HR, ΔISC), key cue search and narrative engagement measures (total and subscales). In the video spoiler group, key cue search frequency showed significant positive associations with narrative presence (*r* = 0.688, *p* = .003) and overall engagement (*r* = 0.561, *p* = .024) and was also positively correlated with emotional engagement (*r* = 0.504, *p* = .046). The association between cues and narrative comprehension was positive but did not reach significance (*r* = 0.447, *p* = .082).

In the complete scene without spoiler group, key cue search frequency showed no significant associations with subjective engagement dimensions. In the chaotic scene without spoiler group, key cue search frequency was significantly positively correlated with Movie ISC-HR (*r* = 0.538, *p* = .047), whereas its correlations with subjective engagement dimensions were not significant. Within the chaotic scene without spoiler group, subjective engagement measures were strongly intercorrelated, including total with attention focus (*r* = 0.841, *p* < .001), narrative presence (*r* = 0.758, *p* =.002), emotional engagement (*r* = 0.630, *p* = .016), and narrative comprehension (*r* = 0.623, *p* = .017). The verbal spoiler group showed no significant associations between key cue search frequency and subjective engagement measures. In the written spoiler group, subjective engagement measures were strongly intercorrelated (e.g., presence–total: *r* = 0.650, *p* = .009; total–emotional engagement: *r* = 0.888, *p* < .001) .

To test whether these patterns differed reliably across groups, Fisher’s *r*—to—*z* tests were conducted (Table 3). The key cue search frequency–presence correlation was significantly stronger in the video spoiler group than in the complete scene without spoiler group (*z* = 2.485,* p* =.013). In addition, the key cue search frequency–total engagement correlation was significantly stronger in the video spoiler group than in the verbal spoiler group (*z* = 2.285, *p* =.022), and the key cue search frequency–emotional engagement correlation also differed between these two groups (*z* = 1.969, *p* =.049).

**Table 3:** Significant differences in correlation coefficients between groups (*p* <.05)

Variable Pairs	Comparison Group	*r*_1_	*r*_2_	*r*_1_ − *r*_2_	sample size (*n*_1_, *n*_2_)	*z*	*p*
Movie ISC-HR vs. Narrative Comprehension	Complete scene without spoiler vs. Video spoiler	− 0.636	0.256	− 0.891	16, 16	− 2.581	0.010^*^
Key cue search Frequency vs. Narrative Presence	Complete scene without spoiler vs. Video spoiler	− 0.129	0.688	− 0.817	16, 16	− 2.485	0.013^*^
Key cue search Frequency vs. Narrative Engagement	Video spoiler vs. Verbal spoiler	0.561	− 0.273	0.834	16, 15	2.285	0.022^*^
Key cue search Frequency vs. Narrative Presence	Video spoiler vs. Verbal spoiler	0.688	− 0.062	0.751	16, 15	2.267	0.023^*^
Movie ISC-HR vs. Key cue search Frequency	Complete scene without spoiler vs. Chaotic scene without spoiler	− 0.281	0.538	− 0.819	16, 14	− 2.172	0.030^*^
Movie ISC-HR vs. Narrative Comprehension	Complete scene without spoiler vs. Written spoiler	− 0.636	0.068	− 0.703	16, 15	− 2.045	0.041^*^
Movie ISC-HR vs. ΔISC-HR	Verbal spoiler vs. Written spoiler	0.725	0.084	0.640	15, 15	2.040	0.041^*^
Key cue search Frequency vs. Emotional Engagement	Video spoiler vs. Verbal spoiler	0.504	− 0.229	0.733	16, 15	1.969	0.049^*^

The significance levels in the table are denoted as follows: ^*^ p <.05

## Discussion

A between-subjects single-factor experimental design was employed to compare the effects of five spoiler types on narrative engagement, frequency of key cues, and ISC-HR in a suspense film. The five groups included a complete scene without spoiler, a chaotic scene without spoiler, and three spoiler modalities (video, verbal, and written). The present findings show that spoiler effects were phase-dependent and facet-specific. Physiological convergence indexed by ISC-HR was more clearly differentiated during spoiler exposure than during movie watching. Self-reported engagement followed a similar profile. Group differences were concentrated in cognitive facets such as narrative understanding and attentional focus, while affective engagement and narrative presence showed little systematic separation. ISC-HR is therefore treated as a complementary index of shared response dynamics across viewers, not a direct measure of subjective immersion. The discussion integrates ISC-HR with self-report and behavioral performance to specify the groups under which different spoiler formats shape audience responses.

### Mechanisms whereby different types of spoilers affect narrative immersion

The ISC-HR results indicate that spoiler types shaped shared physiological response dynamics mainly during spoiler exposure. During spoiler phase, spoiler modality shaped audience synchronization in ISC-HR. In the stimulus-locked, length-matched window aligned to spoiler onset, the video spoiler group showed the strongest ISC-HR synchrony relative to verbal spoiler and written spoiler (*F*_(2,43)_ = 5.34, *p* = .008, *η*^*2*^ = 0.200). The same pattern was also reflected in ΔISC-HR (*F*_(2, 43)_ = 11.911, *p* < .001, *η*^*2*^ = 0.356), indicating that the video spoiler group showed the largest increase in synchrony relative to the comparison reference level reported in the results. The video spoiler group showed a significantly negative ΔISC-HR (*M* =  − 0.045, *SD* = 0.033), whereas the verbal (*M* =  − 0.001, *SD* = 0.045) and written (*M* =  − 0.018, *SD* = 0.039) groups remained close to zero. Predictive coding theory proposes that the brain continuously generates and updates internal models to anticipate incoming sensory input and computes prediction error by comparing expected and actual input (Friston, 2005; Rao & Ballard, 1999). When higher level predictions successfully account for lower level representations, top down feedback reduces lower level activity, which is often described as an explaining away effect (Alink et al., 2010). During spoiler exposure, video spoilers provide time-locked multimodal predictive cues that can align viewers’ expectations and attentional dynamics at the group level, thereby increasing synchrony. During subsequent movie viewing, these predictions are progressively confirmed, prediction error decreases, and processing is adaptively downregulated, leading to a relative decline in synchrony (Keller & Mrsic-Flogel, 2018). Because narrative comprehension relies on hierarchical long range prediction mechanisms, video spoilers may more strongly engage this predictive architecture and thus produce a more pronounced phase-dependent reduction (Caucheteux et al., 2023). In contrast, verbal and written spoilers provide more limited predictive information, and their ΔISC-HR values remained close to zero.

During movie viewing, ISC-HR did not differ significantly across groups (*F*_(4, 71)_ = 0.507, *p* = .731), indicating a phase-specific effect. This pattern is consistent with attentional resource allocation accounts, which suggest that under high task demands, attention becomes tightly focused on goal-relevant information and suppresses processing of task-irrelevant input (Sörqvist et al., 2016). Viewers in the present paradigm had to divide cognitive resources between narrative comprehension and key cue monitoring, and the natural decay of spoiler information in working memory may further reduce the downstream influence of early spoiler exposure on sustained synchrony (Zhang et al., 2011).

From a cognitive perspective, a key advantage of video spoilers may lie in their engagement of multisensory integration networks. Relative to unimodal spoilers, video spoilers provide concurrent auditory and visual cues that can recruit multisensory regions such as the superior temporal cortex (Van Laer et al., 2014). Prior work suggests that multisensory stimuli can elicit higher ISC than unimodal stimuli, with particularly strong effects in multisensory areas (S.S.Cohen & Parra, 2016; Ki et al., 2016; Setti et al., 2023), and may additionally engage action observation and mirroring mechanisms that support group-level alignment (Kilner et al., 2007). By contrast, written spoilers rely primarily on sequential processing within language networks, while verbal spoilers lack stable visual anchors and place greater demands on working memory, both of which may constrain the depth of immersion (Johnson & Rosenbaum, 2015; Nummenmaa et al., 2012).

In the present study, the video spoiler group showed significantly higher narrative comprehension than the chaotic scene group, whereas emotional engagement did not differ reliably across groups, suggesting that spoiler effects were primarily cognitive in nature. The complete no-spoiler group did not show higher engagement than the video spoiler group, which may reflect the role of task complexity. When materials are cognitively demanding, increased load can split attention between narrative integration and cue monitoring and thereby weaken sustained synchrony (Bezdek et al., 2015; Green & Brock, 2000). This pattern diverges from prior conclusions that spoilers reduce immersion while improving task performance, potentially due to differences in task demands and spoiler format (A.L.Cohen et al., 2015). In this account, video spoilers provide a multimodal scaffold that increases predictability at the point of exposure and supports early synchronization, whereas unimodal spoilers offer fewer shared perceptual anchors and therefore yield weaker synchrony (Meel & Vishwakarma, 2021).

### Effects of spoiler types on performance in the key cue search task

The chaotic scene without spoiler group showed the highest key cue search frequency (*M* = 45.643, *SD* = 6.464), significantly exceeding the video spoiler (*M* = 37.312, *SD* = 7.134), verbal spoiler (*M* = 35.400, *SD* = 12.591), written spoiler (*M* = 33.933, *SD* = 8.988), and complete scene without spoiler groups (*M* = 32.062, *SD* = 8.386). The overall effect of group was significant(*F*_(4, 71)_ = 5.003, *p* =.001, *η*^*2*^ = 0.220), and post hoc tests further indicated that the chaotic scene without spoiler group was significantly higher than the complete scene without spoiler, verbal spoiler, and written spoiler groups. This pattern is consistent with a compensatory attention account. When narrative structure is disrupted, viewers lack a coherent framework for predicting and organizing incoming information (Bezdek et al., 2015), which can force a more dispersed and effortful allocation of attention. Under reduced narrative coherence, viewers may shift toward local visual details and increase cue detection as an adaptive attempt to compensate for weakened high-level integration (Sweller, 1988; Zanto & Gazzaley, 2014).

Notably, although the chaotic scene without spoiler group produced the most frequent cue searches, its ISC-HR was not significantly lower than that of other groups. This dissociation suggests that compensatory visual search can increase local task performance without fully restoring shared higher level semantic alignment, consistent with the relative independence of automatic synchrony processes and controlled goal-directed search (Hasson et al., 2008a, 2008b). In contrast, the video spoiler group, while lower than the chaotic scene without spoiler group, remained higher than most other groups, possibly because spoilers reduce uncertainty-related load and free attentional resources that can be reallocated to the monitoring task (Smith et al., 2007). Moreover, advance knowledge of critical information may improve task execution by reducing inefficient search and inference costs, in line with the so-called spoiler paradox (Leavitt & Christenfeld, 2013). The comparatively low performance in the complete scene without spoiler group is consistent with dual-task interference and resource competition (Sweller, 1988). The intermediate performance in the verbal and written spoiler groups may reflect the lower efficiency of unimodal information for building a concrete search template and aligning abstract spoiler content with visual targets, which increases processing demands and reduces search efficiency (Wickens, 2008). Overall, disrupting narrative structure appears to trigger compensatory attention and increased visual search, but this compensation may come at the expense of higher level integration.

### ISC-HR as an auxiliary measure of narrative immersion

In the present study, ISC-HR was used as a complementary physiological index of shared audience response dynamics across viewers. Correlations between ISC-HR and self-reported experience were generally weak, which is consistent with prior work showing that heart rate synchrony is strongly shaped by stimulus-locked processing and attentional alignment, while subjective reports reflect additional person-specific cognitive and affective factors.

In this study, ISC-HR was used as a complementary physiological index of shared audience response dynamics. It provides a physiological perspective on how strongly heart rate fluctuations synchronize across viewers under a common stimulus and is best interpreted alongside self-reported narrative immersion. ISC-HR differed by spoiler modality, with video spoiler showing stronger synchrony than verbal spoiler and written spoiler, consistent with the idea that temporally structured, multimodal input can promote convergence in heart rate fluctuations at the group level (Hammond et al., 2024; Pérez et al., 2021). During movie watching, ISC-HR did not reliably differ across complete scene without spoiler, chaotic scene without spoiler, video spoiler, verbal spoiler, and written spoiler, which suggests that spoiler-related differences in synchrony were phase-specific in the present dataset. Evidence from audio-described film viewing similarly indicates that shared, time-locked sensory information can support comparable physiological response patterns across audiences with different sensory capacities, underscoring the importance of common input structure for synchronization (Hättich & Schweizer, 2020).

To quantify the change in inter-subject synchrony from the spoiler to the movie viewing phase, ΔISC-HR was calculated as a difference metric. A positive value indicates enhanced synchronization during movie watching relative to the spoiler exposure. Verbal spoiler and written spoiler showed lower synchrony at exposure but tended to increase during movie watching as viewers integrated the earlier information with the unfolding narrative. ΔISC-HR therefore complements mean ISC-HR by indicating whether an initial cue-driven alignment is sustained or attenuated across phases, which aligns with work showing that physiological synchrony can peak at structurally salient moments without remaining uniformly elevated across an entire stimulus (Czepiel et al., 2021). Associations between ISC-HR and subjective experience were generally weak, which is compatible with evidence that ISC is more closely tied to attentional alignment than to any single self-report dimension of experience (Liu et al., 2025).

Subjective variables were strongly associated with each other, whereas ISC-HR showed weak and non-significant correlations with self-reported experience, characterized by consistently low coefficients across groups. This pattern is consistent with the view that physiological synchrony and subjective immersion capture related but non-identical aspects of narrative processing (Nummenmaa et al., 2012). Such dissociation may arise from the fact that ISC reflects group-level physiological synchrony primarily driven by immediate stimuli, whereas subjective experience relies on individual differences in cognitive and emotional processing. In the video spoiler group, the correlation between the number of key cues and narrative presence was significantly strengthened, suggesting that visual key cues may indirectly enhance the sense of immersion by improving the efficiency of attentional allocation. This finding provides new evidence for the multidimensional effect of spoilers.

However, this study has several limitations. First, the relatively small sample size (14–16 participants per group) may have limited the ability to detect additional significant differences, particularly given the lack of a significant association between ISC and subjective experience at the group level. Future studies should increase the sample size and investigate whether ISC-HR replicates previously reported correlations. Second, the complexity of spoiler presentation modalities (e.g., multimodal differences between video and verbal spoiler group) should be further examined through controlled experimental manipulation to allow for systematic analysis.

In conclusion, ISC-HR possesses unique value in revealing the influence of different types of spoiler information on audience immersion, serving as a complementary physiological index. It can offer a certain reference for audience immersion as indicated by subjective narrative engagement scale scores, comprehensively assessing the collective response dynamics associated with narrative viewing, especially in evaluating emotional resonance and attentional coordination at the group level. Its partial disconnect from subjective experience indicates that future research should incorporate multimodal physiological data (such as eye tracking and skin conductance) along with dynamic narrative analysis to develop a more comprehensive model for assessing immersion.

### Fundamental role of narrative coherence in the spoiler effect

To distinguish between the separate contributions independent effects of spoiler information and narrative structure on the sense of immersion, this study included a complete scene without spoiler group and a chaotic scene without spoiler group. Across measures, immersive experience and ISC-HR were generally higher in groups that preserved narrative coherence than in the chaotic scene without spoiler group, which supports the view that coherence is a basic pregroup for sustained immersion. The effectiveness of spoilers appears to be constrained by narrative coherence. When viewers subsequently watched a coherent version of the film (complete scene without spoiler, video spoiler, verbal spoiler, and written spoiler), spoiler information modulated processing on top of a stable narrative scaffold. Video spoiler, in particular, provided multimodal, time-locked predictive cues that could be integrated with the later coherent sequence, which helped align expectations and supported early-phase synchronization. In contrast, the chaotic scene without spoiler group lacked a stable temporal and causal structure, leaving viewers with fewer common anchors for constructing a shared situation model and limiting the potential benefits of spoiler information. This pattern is consistent with evidence that disrupted narrative structure weakens coordinated processing and group-level convergence (Hasson et al., 2008a). Under such circumstances, anticipatory guidance is harder to apply, and attention can fragment across disordered events rather than converging on key narrative elements. This mechanism also suggests that moderate use of multi-modal previews or key scene trailers may be most effective when the subsequent material remains structurally coherent, a principle that extends to educational settings where well-organized narratives and advance organizers support comprehension and engagement (Van Laer et al., 2014).

### Differences across dimensions of the narrative engagement scale

An analysis of the four dimensions of the NES revealed significant differences between groups in the dimensions of narrative understanding and attentional focus, while no significant differences were observed in emotional engagement and narrative presence. This pattern suggests that different spoiler types primarily influence cognitive aspects of narrative processing, such as story comprehension and attention allocation, while exerting a more limited impact on emotional resonance and the subjective sense of presence. The video spoiler group achieved the highest scores in narrative understanding, likely owing to the visual and auditory key cues that facilitated smoother processing of the plot (Leavitt & Christenfeld, 2011). In contrast, the chaotic scene without spoiler group scored the lowest in both narrative understanding and attentional focus, indicating the detrimental effect of disrupted narrative coherence on cognitive immersion (A.L.Cohen et al., 2023).

The absence of significant group differences in emotional engagement is likely related to the predictability of the film’s ending and the requirement to prioritize key cue search during viewing, which may have limited the emergence of strong emotional responses (Busselle & Bilandzic, 2009). Compared with prior work suggesting that spoilers can weaken immersion (A.L.Cohen et al., 2015), the present findings indicate that spoiler information did not reduce self-reported immersion under the current task demands. Instead, spoilers appeared to support narrative comprehension and task performance by directing attention toward relevant cues. This interpretation is broadly consistent with the ISC-HR results, which pointed to stronger synchronization during spoiler exposure for formats that provided more structured predictive information. At the same time, the weak correlations between ISC-HR and NES scores underscore that physiological synchrony should be viewed as a complementary index of shared processing rather than a direct proxy for subjective immersion.

### Individual differences in the effect of spoilers

Although this study found that video spoilers generally enhance audiences’ narrative comprehension and sense of immersion, the effects of spoilers can vary significantly across individuals. For instance, “hardcore fans” or highly engaged audiences may regard spoilers as a form of cultural capital, which helps them establish identity within fan communities, enhance their sense of control, and alleviate anxiety caused by narrative uncertainty (Völcker, 2017). Likewise, individuals who prefer psychological preparedness tend to be more receptive to spoilers, as they allow for the construction of mental models, reduce anxiety, and increase attention to character emotions and narrative details (Ellithorpe & Brookes, 2018). Spoilers can also reduce discomfort when watching suspense or horror films, thereby improving the overall viewing experience (Völcker, 2017). From the perspective of cinematic art, frame-by-frame analysis is a professional method for examining audio-visual language and is increasingly being adopted beyond specialist circles. For viewers who value detail or revisit a work multiple times, such as film scholars or literature enthusiasts, spoilers may help establish an analytical framework earlier and thus deepen their understanding and appreciation of narrative structure and cinematographic language (Cohn et al., 2014; Völcker, 2017).

Other audiences may experience spoilers as clearly detrimental. For audiences whose primary motivation for watching lies in suspense and surprise, especially those who prefer mystery or detective genres, spoilers can reduce plot uncertainty and emotional impact, making it harder to experience the expected tension and satisfaction (Li et al., 2022). Similarly, emotionally immersive audiences, who depend on the immediate emotional resonance triggered by plot developments, may find that spoilers disrupt the emotional pacing and weaken the intensity of their engagement (Sukalla et al., 2016). In addition, audiences who prefer simple, linear narratives are more likely to lose interest in exploring the story when exposed to spoilers, as the plot may seem less engaging to them (Völcker, 2017).

Spoilers do not affect all audiences in the same way. For viewers who prefer analytical thinking and place greater emphasis on narrative comprehension, spoilers may improve narrative processing efficiency and even enhance immersion by reducing cognitive load and directing attention more effectively. Conversely, for emotionally driven or suspense-oriented audiences, spoilers may diminish narrative tension and emotional engagement, thereby reducing the sense of novelty and overall satisfaction with the viewing experience. These individual differences underscore the complexity of the spoiler effect, which is neither linear nor one-dimensional but rather emerges from the interplay of viewer motivations, preferences, and contextual factors. Understanding the spoiler effect, therefore, requires analyzing different viewer types and recognizing its inherent variability and context dependence, rather than making overly simplistic assessments of its benefits or drawbacks.

## Conclusion

This study employed a between-subjects single-factor experimental design, incorporating ISC-HR, the NES, and Key cue search task behavior to examine how spoiler formats and narrative coherence shape viewers’ responses to a suspense film. The study reached five key conclusions:

*Video spoilers produced the strongest physiological synchrony during exposure:* The video spoiler group showed significantly higher ISC-HR compared to verbal and written spoiler during the spoiler exposure phase. During movie watching, no significant differences in ISC-HR were observed across any groups. Video spoiler also exhibited a significant negative change from spoiler exposure to movie viewing, indicating stronger initial synchrony followed by adaptive downregulation.

*Chaotic scenes disrupted comprehension but increased visual search:* The chaotic scene group scored lowest on narrative comprehension compared to all other groups yet showed the highest key cue search frequency, significantly exceeding all spoiler conditions and complete scenes. Despite this high search behavior, this group's ISC-HR during movie watching was not significantly different from other groups.

*Video spoilers enhanced cognitive engagement specifically:* Video spoilers achieved the highest narrative comprehension scores, significantly higher than chaotic scenes, verbal spoilers, and complete scenes. Video spoilers also showed higher attention focus than chaotic scenes. However, no significant group differences were found for emotional engagement or narrative presence.

S*poiler effects were phase-specific and modality-dependent:* A significant interaction between experimental phase and spoiler type was found. During spoiler exposure, video spoilers showed significantly higher ISC-HR than verbal spoilers, while no differences existed during movie watching. Only the video spoiler group experienced a significant decline in ISC-HR from spoiler to movie phase.

*ISC-HR and subjective measures captured different aspects:* Correlations between ISC-HR and NES scores were consistently weak and non-significant across all groups. In the video spoiler group, key cue search frequency correlated significantly with narrative presence and total engagement, relationships that were significantly stronger than in other groups. This pattern indicates that physiological synchrony and self-reported engagement reflect distinct but complementary aspects of narrative processing.

## Supplementary Information

Additional file 1.

## Data Availability

The data and analysis code supporting the findings of this study are available on the Open Science Framework (OSF) at: [https://osf.io/jytcp/overview?view_only=8c03fead8656472889d36c0b64329b90]. Key results and methodological details are reported in the, submitted article.

## Appendix A

### Scene 1

Scene 1.1 News of the deaths of several of Gauldel's friends is broadcast on television.

Scene 1.2 Duozaipo and the children look grief-stricken.

Scene 1.3 Due to amnesia, Gauldel doesn't remember these people. A little girl beside him tells him about his relationship with those on TV.

Scene 1.4 Duozaipo bursts into tears.

Scene 1.5 Stunned, Gauldel silently continues eating.

Scene 1.6 Gauldel goes to the beach and kneels to pray, while Duozaipo and the children watch him from behind.

Scene 1.7 Gauldel prays continuously, from day to night, until the tide rises.

Scene 1.8 Gauldel sits alone by the sea, lost in thought.

Scene 1.9 Seeing Ah-Kwai's mark on the pistol, Gauldel decides to take revenge on Mr. Fung, the gang leader.

### Scene 2

Scene 2.1 Mr. Fung is on the phone, sitting in a chair in an open-air square. He hangs up and notices a beautiful woman in a red dress, sporting short curly hair and sunglasses, sitting across from him.

Scene 2.2 Mr. Fung eats his steak.

Scene 2.3 Mr. Fung drinks red wine, smiling at the woman across from him.

Scene 2.4 Mr. Fung flashes his ring to the woman, flaunting his wealth. She smiles and raises her hand. The reflection from her wristwatch shone directly on Mr. Fung.

Scene 2.5: Mr. Fung continued to show off to the beautiful woman, who responded with a smile. Just then, a little girl from a charity approached, asking Mr. Fung to buy a flag sticker. Mr. Fung's subordinate tried to shoo her away, but Mr. Fung signaled to let the little girl put the sticker on him.

Scene 2.6: An increasing number of children appeared before Mr. Fung, all asking him to buy stickers. To save face, Mr. Fung could only repeatedly agree.

Scene 2.7: Once the children had plastered Mr. Fung with stickers, they ran off. Mr. Fung eagerly displayed himself to the beautiful woman across from him, hoping to give her the impression that he was a kind and charitable man. The beautiful woman picked up her coffee and looked up, smiling.

Scene 2.8: With a playful smile, the beautiful woman stood up, ready to leave. A pregnant belly was visible beneath her red dress, revealing that she was, in fact, the "Duozaipo".

Scene 2.9: Realizing he had been played, Mr. Fung gave a self-deprecating laugh.

At this moment, Duozaipo departs.

Scene 2.10 While eating steak, Mr. Fung peels off two stickers from his body and examines them.

### Scene 3

Scene 3.1 Duozaipo and the children leave the open-air plaza, running down the stairs.

Scene 3.2 Duozaipo and the children run to a van where Gauldel and her youngest child are waiting.

Scene 3.3 The other children, standing outside the van, stick the stickers onto Gauldel's pistol.

Scene 3.4 Removing her wig, Duozaipo picks up and soothes her youngest child, telling Gauldel that Mr. Fung has the most stickers.

Scene 3.5 Duozaipo warns Gauldel to be careful, and he agrees.

Scene 3.6 Gauldel gets out of the van, preparing to find Mr. Fung.

### Scene 4

Scene 4.1 Gauldel enters the open-air plaza.

Scene 4.2 Gauldel shouts Mr. Fung's name and raises his pistol, firing a shot.

Scene 4.3 Gauldel fires several shots in the open-air plaza, prompting Mr. Fung's men to defend themselves.

Scene 4.4 Gauldel shoots at Mr. Fung, who is shielded by his men.

Although Gauldel shot at him, his bulletproof vest absorbed the impact.

Scene 4.5 Leaning against the wall, Mr. Fung examined the bullet hole in his vest and cursed angrily. Just then, his men rushed him away from the scene.

### Scene 5

Scene 5.1 Mr. Fung and his men fled into a dimly lit alley.

Scene 5.2 Spotting them, Gauldel resumed firing at Mr. Fung.

Scene 5.3 Mr. Fung and his men continued their escape.

### Scene 6

Scene 6.1 Gauldel attempted to pursue them but was struck by a car while crossing the street.

Scene 6.2 The impact of the car caused Gauldel to suffer a brief period of amnesia. He couldn't recall his mission, but the sight of the pistol and the sticker on it triggered his memory.

Scene 6.3 As Gauldel stood up, preparing to continue chasing Mr. Fung, passersby noticed the gun in his hand and began to whisper among themselves.

Scene 6.4 Gauldel wanted to continue chasing Mr. Fung and his men, but he couldn't clearly remember their faces. He was left with searching the street for people with stickers on them.

Scene 6.5 Mr. Fung and his group hid. From his vantage point, Mr. Fung watched Gauldel search passersby for stickers and began to vaguely understand why.

Scene 6.6 Mr. Fung smirked and transferred all the stickers from his own body onto one of his subordinates.

Scene 6.7 Mr. Fung gestured to a subordinate, signaling him to test the waters.

Scene 6.8 Mr. Fung threw his coat on the ground and fled, now appearing to have no stickers on him. He found a hiding spot.

### Scene 7

Scene 7.1 Mr. Fung's coat was found by Gauldel.

Scene 7.2 In the dimly lit alley, Gauldel shouted Mr. Fung's name, trying to lure him out.

Scene 7.3 Gauldel located Mr. Fung's subordinate by tracking the stickers.

Scene 7.4 Gauldel and Mr. Fung's subordinate exchanged gunfire.

Scene 7.5 The subordinate continued to fire at Gauldel, hitting him in the leg.

Scene 7.6 Mr. Fung watched the scene unfold from under a car. Gauldel shot and killed his two subordinates who were lying in ambush in the dark. By comparing the bullet holes in his coat with those in his assistant's body, he determined that neither of them was Mr. Fung.

### Scene 8

Scene 8.1 From under the car, Mr. Fung witnessed the entire event. With only Gauldel nearby, he drew his handgun and noticed only one bullet remained.

Scene 8.2 Mr. Fung placed the handgun in his suit pants pocket, intending to walk out and blend in as a passerby.

Scene 8.3 As Mr. Fung emerged, Gauldel noticed him.

Scene 8.4 Just then, a gust of wind blew, lifting Mr. Fung's tie and revealing a missing sticker on the back.

Scene 8.5 Realizing what was happening, Gauldel and Mr. Fung exchanged fire. Mr. Fung missed, but Gauldel's shot struck Mr. Fung in the neck.

Scene 8.6 Shot, Mr. Fung collapsed to the ground, clutching his neck.

Scene 8.7: Gauldel questioned Mr. Fung about the coat and demanded he put it on. Under the threat of Gauldel's gun, Mr. Fung had no choice but to comply.

Scene 8.8: Gauldel matched the bullet holes in the coat with those in Mr. Fung's body, confirming that this was the man he sought revenge against.

Scene 8.9: Gauldel shot and killed Mr. Fung before turning and walking alone into the dimly lit street.

## Appendix B

The editing sequence of the Chaotic Scene group is as follows:

Scene 3.3

Scene 8.7

Scene 8.5

Scene 4.5

Scene 5.1

Scene 3.4

Scene 7.3

Scene 1.8

Scene 1.9

Scene 7.2

Scene 7.4

Scene 2.1

Scene 7.1

Scene 3.1

Scene 6.4

Scene 2.4

Scene 3.2

Scene 1.7

Scene 1.2

Scene 1.3

Scene 8.6

Scene 7.6

Scene 7.5

Scene 2.6

Scene 8.4

Scene 3.5

Scene 6.5

Scene 6.3

Scene 1.6

Scene 6.6

Scene 6.2

Scene 8.1

Scene 3.6

Scene 2.2

Scene 4.1

Scene 4.3

Scene 4.4

Scene 2.3

Scene 8.2

Scene 2.10.

Scene 8.9

Scene 4.2

Scene 6.4

Scene 6.7

Scene 6.8

Scene 1.4

Scene 2.8

Scene 1.1

Scene 2.7

Scene 5.2

Scene 5.3

Scene 6.1

Scene 2.5

Scene 1.5

Scene 8.8

Scene 2.9

Scene 8.3

## Appendix C

For completeness, ISC-HR during the spoiler presentation phase was examined among the three spoiler groups (video spoiler, verbal spoiler, and written spoiler). This appendix reports the primary analysis of ISC-HR during the spoiler presentation phase using a stimulus-locked, length-matched window. Specifically, participants’ heart rate time series were aligned to the onset of spoiler exposure (*t* = 0) and ISC-HR was computed within the common 141 s interval shared by the video, verbal, and written spoiler groups. This length-matched computation constitutes the basis for the main results reported in the text; a complementary robustness analysis using the full group-specific duration is provided in Appendix D.

Descriptive statistics indicated that ISC-HR was highest in the video spoiler group (*M* = 0.044, *SD* = 0.032), whereas ISC-HR was lower in the verbal (*M* = 0.002, *SD* = 0.035) and written groups (*M* = 0.021, *SD* = 0.040), suggesting stronger group-level cardiac synchrony when spoilers were delivered in a dynamic audio–visual format (Table 4).

**Table 4:** Descriptive statistics for ISC-HR during spoilering phase

Group	*n*	*M*	*SD*
Video spoiler	16	0.044	0.032
Verbal spoiler	15	0.002	0.035
Written spoiler	15	0.021	0.040

Tukey HSD post hoc tests showed that ISC-HR in the video spoiler group was significantly higher than the verbal group (*p* = 0.006). The video-written group (*p* = 0.325)and the verbal–written contrast (*p* = 0.914) were not significant. Overall, these pairwise results specify that the main group difference in ISC-HR during spoiler exposure was primarily driven by the video modality (Table 5).

**Table 5:** Tukey HSD post hoc comparisons for ISC-HR during spoiler presentation phase

Comparison Group 1	Comparison Group 2	SE	*p*	Cohen's *d*	95% *CI*
Video spoiler	Verbal spoiler	0.016	.006^**^	1.254	[0.011, 0.074]
Video spoiler	Written spoiler	0.016	.325	0.637	[− 0.013, 0.051]
Verbal spoiler	Written spoiler	0.016	.182	− 0.506	[− 0.055, 0.008]

The significance levels in the table are denoted as follows:^**^*p* <.01

To evaluate cardiac synchrony specifically during the spoiler exposure phase, we conducted pairwise comparisons with Bonferroni corrections (see Table 6). The analysis revealed that the video spoiler group elicited significantly different inter-subject synchrony compared to the verbal group (*t* = -2.970, *p* = .006, d = − 1.067), with the video group maintaining a distinct synchronization profile. However, the difference between the video and written groups reached only a marginal level of significance (*t *= − 1.837, *p* = .076), and the comparison between verbal and written modalities was not statistically significant (*t* = 1.115, *p* = .274). These findings suggest that while the video modality significantly diverges from verbal spoilers, its distinction from written spoilers is less pronounced within the length-matched window.

**Table 6:** Bonferroni post hoc comparisons for ISC-HR during spoiler presentation phase

Comparison Group 1	Comparison Group 2	*t*	*p*	Cohen's *d*	95% *CI*
Video spoiler	Verbal spoiler	− 2.970	0.006^****^	− 1.067	[− 1.824, − 0.311]
Video spoiler	Written spoiler	− 1.837	0.076	− 0.660	[− 1.385, 0.064]
Verbal spoiler	Written spoiler	1.115	0.274	0.407	[− 0.316, 1.131]

The significance levels in the table are denoted as follows:^****^* p* <.01

The length-matched, stimulus-locked results in Appendix C establish the primary evidence that spoiler modality affects ISC-HR during spoiler exposure, with the video spoiler group showing the strongest synchrony; Appendix D further demonstrates that this overall pattern is consistent when ISC-HR is recomputed using the full group-specific duration.

## Appendix D

Because the three spoiler materials differed in duration (video: 4 min 57 s; verbal: 2 min 21 s; written: 2 min 40 s), this appendix reports a robustness check in which ISC-HR was recomputed using the full available duration within each spoiler group (group-specific full length). This analysis complements the main results in the text, which were based on a stimulus-locked, length-matched window to ensure cross-group comparability.

Descriptive statistics (Table 7) indicated that ISC-HR was highest in the video spoiler group (M = 0.108, SD = 0.078), followed by the verbal spoiler group (M = 0.010, SD = 0.059), with the written spoiler group showing the lowest synchrony (M = 0.020, SD = 0.042). This ordering is consistent with the pattern reported in the main analysis.

**Table 7:** Descriptive statistics for ISC-HR during full length spoilering phase

Group	*n*	*M*	*SD*
Video spoiler	16	0.108	0.078
Verbal spoiler	15	0.010	0.059
Written spoiler	15	0.020	0.042

The one-way ANOVA (Table ﻿8) showed a significant overall effect of spoiler presentation mode on ISC-HR under the full length computation, *F*_(2,43)_ = 11.996, *p *< 0.001, *η*^2 ^= 0.357, indicating that ISC-HR differed across the three spoiler groups in this robustness analysis.

**Table 8:** One-way ANOVA results for ISC-HR during full length spoiler presentation phase

Source	*SS*	*df*	*MS*	*F*	*p*	*η*^*2*^
Between groups	0.091	2	0.046	11.996	<.001^***^	0.357
Within groups	0.164	43	0.004			
Total	0.255	45				

The significance levels in the table are denoted as follows:^***^*p* <.001

As shown in Table 10, Tukey HSD post hoc comparisons indicated that the video spoiler group produced significantly higher ISC-HR than both the verbal spoiler group (*p* < 0.001) and the written spoiler group (*p* = 0.001). In contrast, the difference between the verbal and written groups was not significant after Tukey adjustment (*p* = 0.904). These findings suggest that the group-level synchronization advantage observed during the spoiler phase was primarily driven by the video modality, whereas the two non-video modalities yielded comparable ISC-HR levels.

**Table 9:** Tukey HSD post hoc comparisons for ISC-HR during full length spoiler presentation phase

Comparison Group 1	Comparison Group 2	SE	*p*	95% *CI*	Cohen's *d*	Hedges’ *g*
Video spoiler	Verbal spoiler	0.028	< 0.001^***^	[0.044, 0.152]	1.416	1.379
Video spoiler	Written spoiler	0.028	0.001^**^	[0.035, 0.142]	1.400	1.363
Verbal spoiler	Written spoiler	0.028	0.904	[− 0.045, 0.064]	− 0.195	− 0.190

The significance levels in the table are denoted as follows:^**^*p* <.01,^***^*p* <.001

Although spoiler durations differed across groups, the full length analysis showed that the video spoiler group yielded the strongest ISC-HR and was significantly higher than both the verbal and written groups, mirroring the pattern observed in the main stimulus-locked, length-matched analysis. These convergent findings support the appropriateness of using the time-aligned, length-matched approach for ISC-HR estimation in the primary results.

## Declarations

### AI use statement

During manuscript preparation, the authors used a generative AI tool to assist with drafting portions of the data-analysis code, including code used to generate statistical outputs, tables, and figures, and to support language editing and wording refinement. The study concept, experimental design, and hypotheses were independently developed by the authors. All analytical decisions, code verification, interpretation of results, and final manuscript content were reviewed, finalized, and approved by the authors, who take full responsibility for the article.
